# A Gamma-Herpesvirus Glycoprotein Complex Manipulates Actin to Promote Viral Spread

**DOI:** 10.1371/journal.pone.0001808

**Published:** 2008-03-19

**Authors:** Michael B. Gill, Rachel Edgar, Janet S. May, Philip G. Stevenson

**Affiliations:** Division of Virology, Department of Pathology, University of Cambridge, Cambridge, United Kingdom; Karolinska Institutet, Sweden

## Abstract

Viruses lack self-propulsion. To move in multi-cellular hosts they must therefore manipulate infected cells. Herpesviruses provide an archetype for many aspects of host manipulation, but only for alpha-herpesviruses in is there much information about they move. Other herpesviruses are not necessarily the same. Here we show that Murine gamma-herpesvirus-68 (MHV-68) induces the outgrowth of long, branched plasma membrane fronds to create an intercellular network for virion traffic. The fronds were actin-based and RhoA-dependent. Time-lapse imaging showed that the infected cell surface became highly motile and that virions moved on the fronds. This plasma membrane remodelling was driven by the cytoplasmic tail of gp48, a MHV-68 glycoprotein previously implicated in intercellular viral spread. The MHV-68 ORF58 was also required, but its role was simply transporting gp48 to the plasma membrane, since a gp48 mutant exported without ORF58 did not require ORF58 to form membrane fronds either. Together, gp48/ORF58 were sufficient to induce fronds in transfected cells, as were the homologous BDLF2/BMRF2 of Epstein-Barr virus. Gp48/ORF58 therefore represents a conserved module by which gamma-herpesviruses rearrange cellular actin to increase intercellular contacts and thereby promote their spread.

## Introduction

Viruses are inherently non-motile. They must therefore rely for their movement on manipulating infected cells. In lytic infection, this means maximizing the number of uninfected neighbours each infected cell contacts and the distance each round of replication travels. Both parameters depend on cell shape. Vaccinia virus promotes its spread by inducing actin tail formation with A36R [1], [2]. Herpesviruses can disseminate systemically as latent genomes-gamma-herpesviruses hitchhike on normal lymphocyte circulation [3]-but they must still move between their latency reservoir and an epithelial surface.

Most information on herpesvirus spread comes from the neurotropic alpha-herpesviruses. For example, the Herpes simplex virus gE/gI complex promotes viral spread across tight junctions [4], and the Pseudorabies virus US3 kinase induces large, tubulin-filled cytoplasmic extensions [5] akin to the axons normally used to move between sites of lytic and latent infection. Very little is known about the intercellular spread of lymphotropic gamma-herpesviruses. This reflects that Epstein-Barr virus (EBV) and the Kaposi's Sarcoma-associated Herpesvirus (KSHV) both have narrow species tropisms and are difficult to propagate lytically *in vitro*. However, their lytic genes are generally conserved in other family members, which can therefore tell us how EBV and KSHV are likely to work. Murine gamma-herpesvirus-68 (MHV-68) [6], [7] provides a particularly accessible model. It disseminates *in vivo* without an obvious cell-free viraemia [8], suggesting that direct intercellular spread plays an important role in pathogenesis. Indeed, genes involved in intercellular spread [9], [10] seem to contribute more to *in vivo* replication than those involved in cell-free virion binding [11] or release [12].

We have previously identified ORFs 27 and 58 as important for intercellular MHV-68 spread [9], [10]. Both are conserved in other gamma-herpesviruses. ORF27 encodes a heavily glycosylated type II transmembrane protein (gp48) that is abundantly expressed on infected cell plasma membranes. ORF58 encodes a multi-membrane spanning protein, without which gp48 is retained in the endoplasmic reticulum. Transfected ORF58 localizes to the endoplasmic reticulum and trans-Golgi network, but co-transfected gp48 and ORF58 form a complex and reach the cell surface. How gp48 and ORF58 promote intercellular viral spread is unknown. The ORF58 homolog of EBV, BMRF2, contains an integrin binding motif that has been implicated in virion adhesion to basolateral epithelial surfaces [13]; the equivalent loop of the MHV-68 ORF58 also binds to cells [10]. However, EBV and MHV-68 each has at least 3 other cell-binding proteins. So while intercellular spread must involve binding, this seems unlikely to be rate-limiting. Analogy with vaccinia virus would suggest that cell shape is more important.

A cell's shape is determined largely by its cytoskeleton. Thus, different patterns of actin polymerization generate structures such as filopodia, lamellopodia or membrane ruffles under the control of Rho GTPases [14]. The best-known of these are RhoA, Rac1 and Cdc42 [15]. Lamellipodia and membrane ruffles require Rac1; stress fibres and focal adhesions require RhoA; and filopodia require Cdc42. As with many important cellular processes, pathogens have evolved ways to polymerize actin for their own ends [16], [17]. Indeed, the exaggerated behaviour of pathogens often provides a convenient route to understanding the underlying cell biology. Detailed descriptions of viral actin manipulation have so far been limited to poxviruses. Other viruses may have subtly different goals and achieve them in subtly different ways [18]. Our aim here was to understand how the MHV-68 gp48/ORF58 complex promotes its spread. Our attention was drawn to actin as an important player by gp48 decorating fine extensions of the infected cell plasma membrane [9]. These membrane fronds were found to be actin-based and RhoA-dependent. Time-lapse confocal imaging showed the whole membrane to be highly dynamic, with gp48-dependent fronds reaching out to uninfected cells and supporting virion movement. Actin polymerization therefore appeared to be a major part of the mechanism by which gp48 and ORF58 promote MHV-68 spread.

## Results

### MHV-68 remodels the infected cell plasma membrane

**Figure 1:**
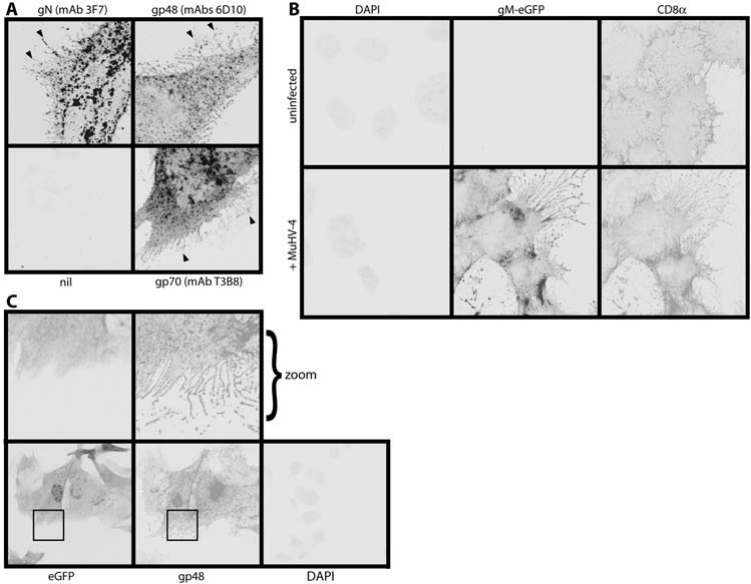
Plasma membrane remodelling by MHV-68. A. NIH-3T3 cells were infected (1 p.f.u./cell, 16 h) with wild-type MHV-68, then fixed, permeabilized and stained for viral glycoproteins. Positive staining appears black or grey. gN, gp48 and gp70 could each be seen on frond-like extensions of the infected cell plasma membrane (arrows). nil = secondary antibody only. B. NIH-3T3 cells stably expressing human CD8α were infected (1 p.f.u./cell, 16 h) or not with MHV-68 expressing eGFP-tagged gM. The cells were then fixed, permeabilized and stained for CD8α. Nuclei were counter-stained with DAPI. eGFP fluorescence was visualized directly. A similar MHV-68 induction of membrane fronds was seen using a lipophilic dye to stain the plasma membrane (data not shown). C. NIH-3T3 cells were infected (1 p.f.u./cell, 16 h) with MHV-68 expressing free eGFP, then fixed and stained for gp48 with mAb T8H3. eGFP fluorescence was visualized directly. Nuclei were counter-stained with DAPI. The zoomed images correspond to the boxed regions in the upper panels.

The MHV-68 gp48 accumulates on fine protrusions that reach out from infected cells [9]. Close examination revealed that other virion glycoproteins such as gN and gp70, although more abundant on intracellular membranes, also occupied plasma membrane protrusions (Figure 1A). These were common to MHV-68-infected NIH-3T3 cells, BHK-21 cells, NMuMG cells and primary embryonic fibroblasts. Figure 1 shows NIH-3T3 fibroblasts and Figure S1 shows NMuMG epithelial cells. Comparing the distribution of transfected CD8α between infected and uninfected cells (Figure 1B) made it clear that the membrane protrusions were induced by MHV-68. They were long, branched and extremely fine, excluding cytoplasmic eGFP (Figure 1C). They also appeared to be fragile, as fragmented protrusions were abundant around the periphery of each infected cell. Thus, they were distinct from filopodia, lamellipodia or actin tails. We refer to them as membrane fronds.

**Figure 2:**
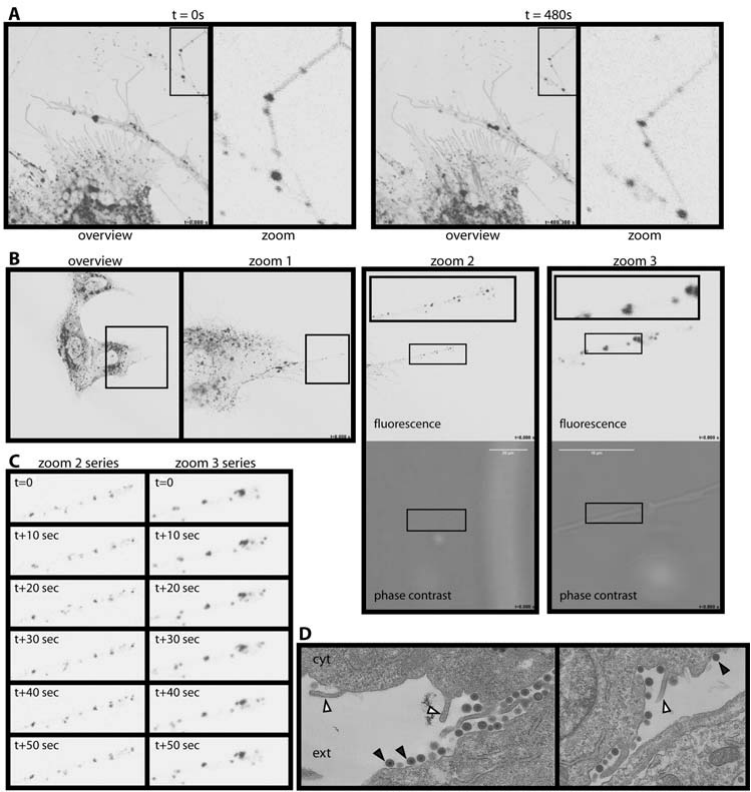
Relationship between membrane fronds and virions. A. BHK-21 cells infected with MHV-68 expressing eGFP-tagged gM (1 p.f.u./cell, 16 h) were examined by time-lapse confocal microscopy. The eGFP signal appears as black/gray. Each zoomed image corresponds to the boxed region of the corresponding overview. The punctate fluorescence of distal membrane fronds is seen to change with time. See also Movies S1 and S2. B. BHK-21 cells were infected with gM-eGFP-tagged MHV-68 as in A. Time-lapse imaging then focussed on a single membrane process. Zoom 1 is the boxed region in the overview; zoom 2 is the boxed region of zoom 1, with its boxed region shown as a zoomed inset. Zoom 3 shows a region equivalent to the central part of zoom 2 at ×5 greater magnification. Again, its boxed region is shown as a further zoomed inset. The complete sets of zoom 2 and zoom 3 pictures make up Movies S3 and S4. C. Stills from Movies S3 and S4, which correspond to the zoom 2 and zoom 3 images in B, show the variation in eGFP^+^ dot distribution at 10sec intervals. D. BHK-21 cells were infected with MHV-68 (1 p.f.u./cell, 16h) then fixed and processed for transmission electron microscopy. cyt = cytoplasm; ext = extracellular. Closed arrows show virions. Open arrows show the bases of membrane fronds.

We viewed infected cells in real time using MHV-68 with eGFP-tagged gM (Figure 2, Movies S1 and S2). Because gM is an abundant virion glycoprotein, individual tagged virions can be seen by confocal microscopy [19], [20]. gM-eGFP accumulated in discrete dots and vesicles (Figure 2A, 2B) which moved rapidly and bi-directionally along the membrane fronds. Tracking a single membrane process (Figure 2C, Movies S3 and S4) showed that the eGFP^+^ dots changed noticeably in distribution over 10 second intervals. The infected cell plasma membrane was also highly dynamic (Movies S1, S2 and S5). In comparison with the motile proximal membrane, most of the distal fronds were fixed, presumably because they adhered to plastic. Tension generated between the two by their relative movement explained the abundance of broken fronds.

The MHV-68 gN and gM form a disulfide-linked complex that homes to the trans-Golgi network [21]. The gM-eGFP^+^ dots on distal membrane fronds (Figure 2), like gN in the same site (Figure 1A), were therefore likely to be exiting virions. EGFP^+^ vesicles could be seen on membrane processes leading up to the fronds (Movie S2), but the eGFP^+^ dots on distal fronds did not match obvious features on the phase contrast images (Figure 2B) because virions are not visible at this magnification. Each dot was asymmetrically placed and mobile around the axis of each frond (Movie S4), suggesting that the virions were on the outside. Electron microscopy (Figure 2D) showed abundant MHV-68 virions on infected cell plasma membranes, but not under the plasma membrane as would be required for entry into the membrane fronds. The virions were also larger in diameter than processes extending out from the plasma membrane. Finally, gp150 was accessible on the fronds without permeabilization (Figure S2). The virions were therefore attached to the outside of the membrane fronds.

### Infected cell membrane remodelling requires gp48

**Figure 3:**
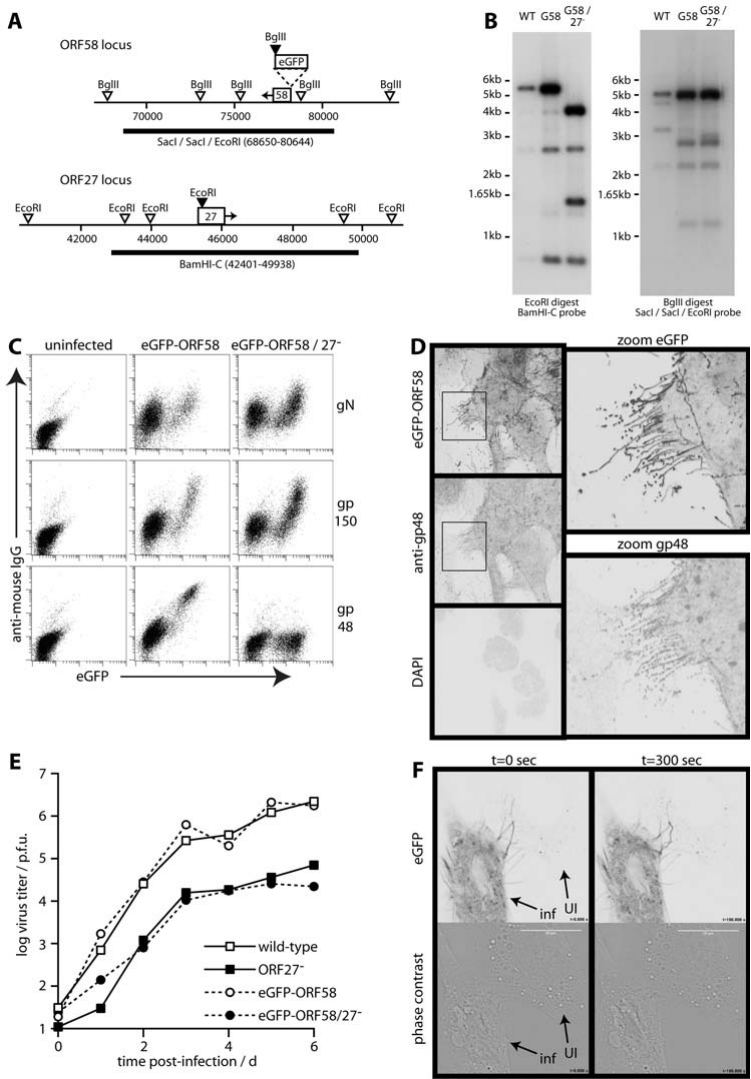
eGFP-tagged ORF58 decorates the membrane fronds. A. Schematic diagram of the MHV-68 ORF58 and ORF27 loci. Filled arrowheads show restriction sites created by eGFP tagging ORF58 or disrupting ORF27. Open arrowheads show genomic restriction sites. The thick lines show probe locations. B. Southern blots of wild-type (WT), eGFP-tagged ORF58 (G58) and ORF27-deficient eGFP-tagged ORF28 (G58/27^−^) viruses. The *Eco*RI digest plus *Bam*HI-C probe gave predicted wild-type and G58 virus fragments of 5611bp, 2703bp, 744bp and 1342bp (difficult to see because of limited overlap with the probe). The *Eco*RI-restricted oligonucleotide introduced into ORF27 at genomic co-ordinate 45480 converted the 5611bp band to 4075bp plus 1536bp. The *Bgl*II digest plus *Sac*I/*Sac*I/*Eco*RI probe gave predicted WT fragments of 5300bp, 5165bp, 2294bp and 3379bp. The eGFP insertion just upstream of ORF58 converted the 3379 band to 1193bp+2916bp. C. BHK-21 cells were left uninfected or infected (0.5 p.f.u./cell, 16 h) with ORF27^+^ or ORF27^−^ versions of the eGFP-ORF58 virus. The cells were then stained for gN (mAb 3F7), gp150 (mAb T1A1) or gp48 (mAb T8H3), each with phycoerythrin-conjugated goat anti-mouse IgG pAb. The data are from 1 of 3 equivalent experiments. D. NIH-3T3 cells were infected with eGFP-ORF58 MHV-68 (1 p.f.u./cell, 16 h), then fixed, permeabilized and stained for gp48 with mAb 6D10 plus Alexa568-conjugated goat anti-mouse IgG pAb. EGFP fluorescence was visualized directly. Nuclei were counterstained with DAPI. The zoomed images correspond to the boxed regions in the left-hand panels. The data are from 1 of 5 equivalent experiments. E. BHK-21 cells were infected (0.01 p.f.u./cell) with ORF27^+^ or ORF27^−^ versions of either untagged (wild-type) or eGFP-ORF58-tagged MHV-68. Virus titers were determined by plaque assay at the times indicated. F. NIH-3T3 cells were infected with eGFP-ORF58 MHV-68 (1 p.f.u./cell, 16h) then examined by time-lapse confocal microscopy. An infected (inf) cell is shown next to an uninfected (UI) or only recently infected neighbour to demonstrate eGFP^+^ fronds reaching from one to the other. Movie S5 shows the difference between these cells in shape and motility. The findings were typical of >100 cells examined.

The increase in cell reach and surface area generated by the membrane fronds suggested that they might play a role in virus spread. We therefore hypothesized that the intercellular spread proteins encoded by ORFs 27 and 58 might help to form the fronds. In order to identify ORF58 in virus-infected cells, we tagged it with N-terminal eGFP, as done previously for transfected cells [10]. Southern blots confirmed the predicted genomic structures of gp48^+^ and gp48^−^ versions of this tagged virus. (Figure 3A, 3B). EGFP-tagged ORF58 supported (Figure 3C) and co-localized with (Figure 3D) gp48 expression at the cell surface. Growth curves showed a gp48-dependent spread deficit similar to that seen on a wild-type background (Figure 3E). EGFP tagging therefore did not compromise ORF58 function. Time-lapse imaging showed eGFP-ORF58^+^ processes reaching out from infected cells and contacting their uninfected neighbours (Figure 3F, Movie S5), consistent with a connection between gp48/ORF58 expression, membrane frond formation, and intercellular spread.

**Figure 4:**
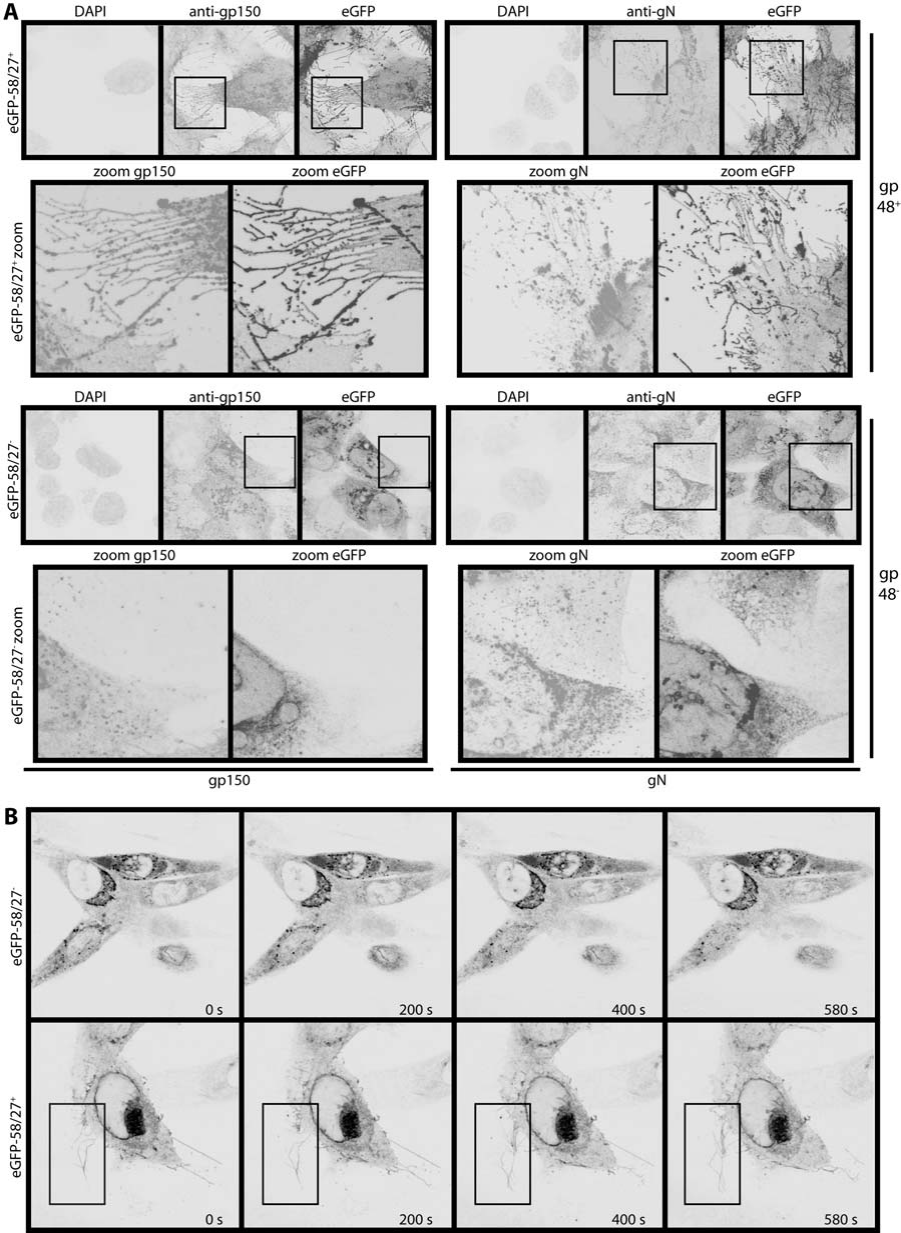
MHV-68-induced membrane changes are gp48-dependent. A. NIH-3T3 cells were infected (1 p.f.u./cell, 16 h) with gp48^+^ (upper set of images) or gp48^−^ (lower set of images) versions of eGFP-ORF58 MHV-68, then fixed, permeabilized and stained for gp150 (mAb T1A1, left set of images) or gN (mAb 3F7, right set of images). Each mAb was visualized with Alexa568-conjugated goat anti-mouse IgG pAb, eGFP was visualized directly, and nuclei were counterstained with DAPI. The zoomed images correspond to each boxed region above. B. NIH-3T3 cells were infected with ORF27^−^ or ORF27^+^ eGFP-ORF58 viruses as in A, then visualized by time-lapse confocal microscopy. The boxed region highlights eGFP^+^ membrane fronds growing away from the ORF27^+^ infected cell surface. Times are indicated in each panel. The complete image sets make up Movies S6 and S7.

While the eGFP-ORF58 of gp48^+ ^MHV-68 (Figure 4A) outlined fronds reaching out from infected cells, the eGFP-ORF58 of gp48^−^ MHV-68 (Figure 4B) did not. This was not surprising, as ORF58 transit from the trans-Golgi network to the plasma membrane of transfected cells is gp48-dependent [10]. However, neither were gN or gp150 seen on fronds without gp48 (Figure 4A), and they did not depend on gp48 for transport (Figure 3C). It therefore appeared that without gp48, the fronds did not form. (Flow cytometry probably shears off the fragile distal fronds and would therefore measure mainly proximal membrane glycoprotein expression.) Time-lapse imaging showed that without gp48, there was much reduced contact between infected and uninfected cells (Figure 4C, Movies S6 and S7). For example, the boxed region in Figure 4C outlines eGFP^+^ fronds growing out from the gp48^+^ infected cell surface to contact a neighbouring uninfected cell (Movie S6), whereas gp48^−^ infected cells remained quite separate from their uninfected neighbours (Movie S7).

### The infected cell membrane protrusions contain actin but not tubulin

**Figure 5:**
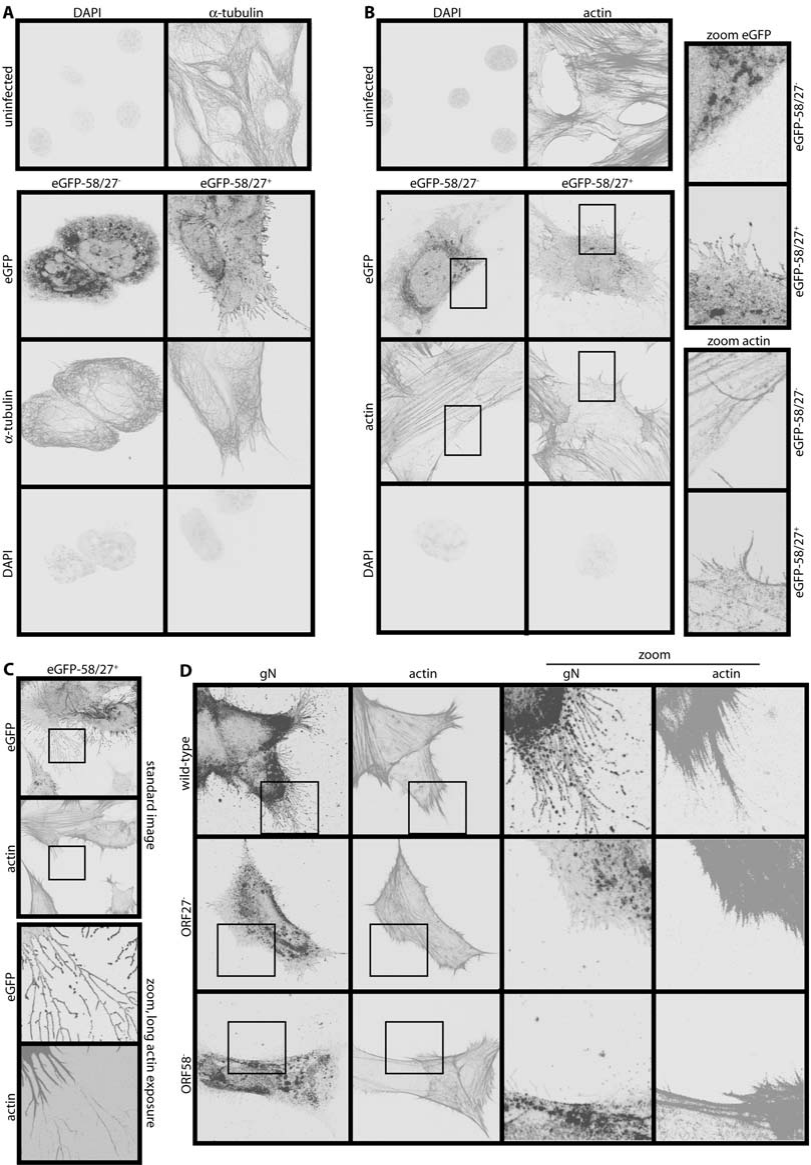
MHV-68-induced membrane fronds contain actin but not tubulin. A. NIH-3T3 cells were left uninfected or infected (1 p.f.u./cell, 16 h) with either ORF27^−^ or ORF27^+^ eGFP-ORF58 MHV-68. They were then fixed, permeabilized and stained for α-tubulin. EGFP fluorescence was visualized directly. Nuclei were counterstained with DAPI. B. NIH-3T3 cells were infected or not as in A, then fixed, permeabilized and stained for actin with Alexa568-conjugated phalloidin. EGFP fluorescence was visualized directly and nuclei were counterstained with DAPI. The zoomed images correspond to the boxed regions. They show coincident membrane fronds and actin spikes with ORF27^+^ infection and neither with ORF27^−^ infection. C. NIH-3T3 cells were infected (1 p.f.u./cell, 16 h) with ORF27^+^ eGFP-ORF58-tagged MHV-68 and stained for actin as in B, but with longer exposure times for the zoomed images (which correspond to the boxed regions above) to show the very fine actin cores of the distal membrane fronds. D. NIH-3T3 cells were infected (1 p.f.u./cell, 16 h) with untagged wild-type, ORF27^−^ or ORF58^−^ MHV-68. The cells were then fixed, permeabilized and stained for gN with mAb 3F7 plus Alexa488-conjugated goat anti-mouse IgG pAb, and for actin with Alexa568-conjugated phalloidin.

Plasma membrane shape is set by the cytoskeleton. Therefore in order to understand how MHV-68 induced membrane fronds, we examined the distribution of actin and tubulin in infected cells. The tubulin network appeared quite normal. Tubulin entered larger membrane processes but not the extensive distal fronds (Figure 5A), and showed no obvious difference between gp48^+^ and gp48^−^ infections. Actin, however, was very different between infected and uninfected cells and between gp48^+^ and gp48^−^ viruses (Figure 5B). MHV-68-infected cells lost the prominent actin stress fibres of uninfected control cells, and rearranged actin in the pattern of their membrane fronds. Because the distal fronds were so fine, their actin cores were seen best at high magnification with extended exposure times (Figure 5C). A marked difference in actin organization between wild-type, ORF27^−^ and ORF58^−^ infections was confirmed with untagged viruses (Figure 5D). The formation of actin spikes, like the formation of membrane fronds, therefore required both ORF58 and gp48, and frond formation was presumably driven by actin polymerization.

### Gp48 reorganizes actin from the plasma membrane; ORF58 is required for gp48 transport

**Figure 6:**
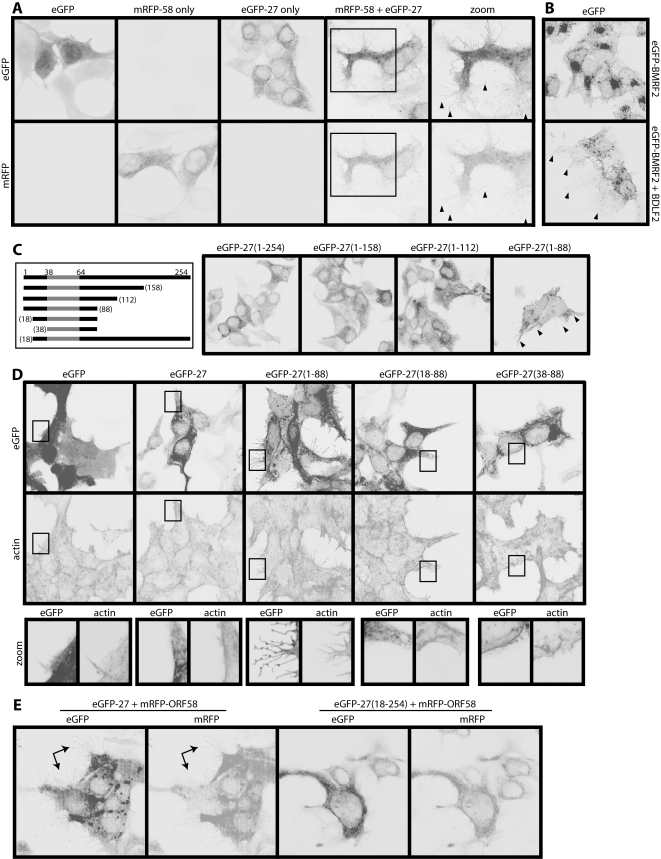
ORF27 and ORF58 are sufficient to induce membrane fronds. A. 293T cells were transfected with expression plasmids for eGFP, mRFP-tagged ORF58 (mRFP-58), eGFP-tagged ORF27 (eGFP-27), or mRFP-58 plus eGFP-27. Red and green fluorescence signals were examined 48 h later. The zoomed images match the boxed region in the mRFP-58+eGFP-27 transfection. Arrowheads show membrane fronds. B. 293T cells were transfected with an expression plasmid for eGFP-tagged BMRF2 (EBV ORF58 homolog), with or without an expression plasmid for BDLF2 (EBV ORF27 homolog). 48 h later, the eGFP signal was visualized. Arrowheads show membrane fronds, which were not seen with either plasmid alone. Note also eGFP-BMRF2 redistribution following BDLF2 transfection, much like that seen with ORFs 27 and 58. C. ORF27 truncations, each tagged with eGFP (our mAbs only recognize full-length ORF27) were transfected into 293T cells. 48 h later, the cells were fixed and eGFP fluorescence visualized. The numbers correspond to amino acid residues of the full-length protein (gp48). The gp48 C-terminal domain is extracellular. The shaded region corresponds to its transmembrane domain. The arrows show membrane fronds, which were prominent only with the eGFP-27(1-88) mutant. Note also the redistribution of eGFP fluorescence. D. 293T cells were transfected with different eGFP-tagged ORF27 truncation mutants and 48h later fixed, permeabilized and stained for actin with Alexa568-conjugated phalloidin. EGFP fluorescence was visualized directly. The boxed region for each transfection is shown in the zoomed images below. E. 293T cells were transfected with mRFP-tagged ORF58 plus either full-length or N-truncated ORF27. 48 h later, eGFP (ORF27) and mRFP (ORF58) fluorescence signals were visualized. The arrows show membrane fronds with the full-length form. These were never seen with the cytoplasmic tail truncation mutants.

Having established that ORFs 27 and 58 were necessary for MHV-68-induced actin rearrangement and membrane remodelling, we next tested whether they were sufficient. Transfecting 293T cells with either ORF27 or ORF58 alone had little effect, but co-transfecting ORF27 and ORF58 induced fronds similar to those of infected cells (Figure 6A). Membrane fronds were also induced by the ORF27 and ORF58 homologs of EBV, BDLF2 and BMRF2 (Figure 6B). Thus, despite these proteins being quite divergent in sequence, their membrane remodelling function was conserved.

In order to define key functional regions of gp48/ORF58, we made a series of gp48 truncation mutants (Figure 6C). Suprisingly, truncating the gp48 extracellular domain (eGFP-27(1-88)) allowed it to reach the plasma membrane and induce membrane fronds independent of ORF58. In contrast to this releasing effect of C-terminal truncation, N-terminal truncation abolished completely the induction of fronds or actin spikes (Figure 6D, 6E). The gp48 cytoplasmic tail was therefore critical for actin rearrangement, whereas its extracellular domain was redundant and acted mainly to make the rearrangement ORF58-dependent. Without a need for gp48 transport, ORF58 was dispensible.

**Figure 7:**
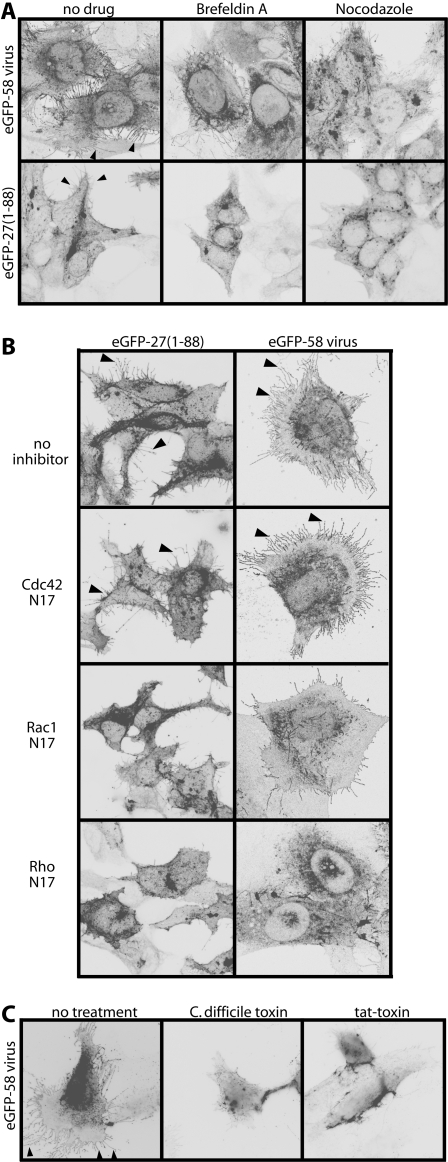
Gp48 induces membrane fronds via RhoA. A. NIH-3T3 cells were either infected with eGFP-tagged ORF58 MHV-68 (1 p.f.u./cell) or transfected with the eGFP-tagged C-truncated ORF27(1-88) mutant. 18h later, the cells were treated or not for 1h with Brefeldin A or nocodazole. They were then fixed and examined for eGFP fluorescence. The arrowheads show membrane fronds on the cells without drug treatment. B. NIH-3T3 cells were either infected with eGFP-tagged ORF58 MHV-68 (1 p.f.u./cell) or transfected with the eGFP-tagged C-truncated ORF27(1-88) mutant. 4 h later they were transfected or not with dominant negative inhibitors of Cdc42 (Cdc42N17), Rac1 (Rac1N17) or RhoA (RhoN19). After a further 18h, the cells were fixed and examined for membrane fronds based on eGFP fluorescence. The arrows show fronds on the cells without inhibitors or with only Cdc42 inhibited. C. NIH-3T3 cells were infected with eGFP-ORF58 tagged MHV-68 (1 p.f.u./cell, 16 h) then exposed to either *C.difficile* toxin or its active moiety fused to an HIV tat transporter peptide. 4 h later, the cells were fixed and examined for membrane fronds based on eGFP fluorescence. The arrowheads show fronds on the cells without toxin treatment.

Further evidence that gp48 must reach the plasma membrane to function came from drug treatments (Figure 7A). Exposing MHV-68-infected cells or eGFP-27(1-88)-transfected cells to Brefeldin A or nocodazole for just 1h was sufficient to reduce significantly their membrane fronds. Brefeldin A blocks glycoprotein export from the ER; nocodazole collapses the microtubule network. Since neither treatment was started until gp48 was already in place, these results indicated that the gp48/actin interaction is highly dynamic, depending on continued gp48 export. This was consistent with the enormous increase in cell surface area created by the fronds and with their tendency to break off: plasma membrane gp48 would have to be constantly replenished.

### Actin rearrangement by gp48 requires Rho GTPase function

Although the gp48-induced fronds conformed to none of the archetypal actin-based membrane protrusions, it still seemed likely that their formation involved GTPase signalling. We therefore used dominant negative mutants to establish whether RhoA, Rac1 or Cdc42 were involved (Figure 7B). Again we tested both MHV-68-infected and eGFP-27(1-88)-transfected cells. Dominant negative Cdc42 had no effect; dominant negative Rac1 was modestly inhibitory; and dominant negative RhoA was strongly inhibitory. An important role for RhoA was confirmed by treating MHV-68-infected cells with *Clostridium difficile* toxin or with the active component of this toxin linked to an HIV tat-derived transport motif. Both treatments markedly reduced frond formation. Thus, it appeared that the gp48 cytoplasmic tail uses GTPase signalling, predominantly that of RhoA, to change the shape of infected cells.

## Discussion

Each cell of a complex organism only ever displays a fraction of its possible functions. Viruses undo the normal restraints on cell behaviour, and can thereby reveal new aspects of cell biology. MHV-68 remodelled the plasma membrane of infected cells into multiple, fine, branched fronds that reached out to neighbouring cells. This inappropriate sociability of infected cells was driven by the cytoplasmic tail of gp48, an MHV-68 glycoprotein involved in intercellular virus dissemination. The fronds were quite unlike vaccinia virus-induced actin tails, which are robust and seem best suited to pushing virions out against resistance [2]. The MHV-68-induced fronds instead enhanced intercellular contacts by increasing cell surface area and reach. Also, they did not form underneath virions like actin tails, but created a scaffold that captured secreted virions and gave direction to their subsequent movement. The MHV-68-induced fronds are perhaps analogous to the filopodial bridges formed by retroviruses [22]. However, they appeared to be much more extensive, and unlike filopodial bridges were highly branched. Frond formation required RhoA, implying that gp48 recruits RhoA-based adaptors, much as the vaccinia virus A36R recruits Src family kinases and Nck [23], [24]. Thus, analogous cell signalling pathways can be used by different viruses for quite different functional ends.

Mature herpes virions bud into intracellular membranes and therefore leave cells via exocytic vesicles [25]. The nature of these vesicles remains poorly defined. They may well be heterogenous. Ultimately, however, they must all fuse with the plasma membrane to release their virions into the extracellular space. Such an exit pathway sequesters virions from cytoplasmic actin. Thus, although MHV-68 virions contain some gp48 [9], that manipulating actin must be in the limiting membrane of secretory vesicles. As gp48 had to reach the plasma membrane to function, it presumably initiates actin polymerization when the virion-containing vesicles fuse with the plasma membrane. The gp48 cytoplasmic tail would then be correctly orientated to interact with RhoA-dependent signalling.

The ORF58 independence of C-truncated ORF27 for both transport to the cell surface and actin polymerization implied that what ORF58 contributes to frond formation is gp48 transport. This would allow ORF58 to control frond formation in infected cells, and since ORF58 also drives MHV-68 secondary envelopment [10], it can co-ordinate virion maturation and dissemination. EGFP-ORF58^+^ vesicles were often seen under the plasma membranes of MHV-68-infected (Figure 2A, Movies S1 and S2) and ORF58/ORF27 co-transfected cells (Figure 6E). These vesicles presumably go on to fuse with the plasma membrane. In infected cells this would simultaneously release virions into the extracellular space and initiate gp48-dependent actin polymerization. The extracellular virions would then be collected on nascent membrane fronds and carried to neighbouring uninfected cells. Apical epithelial fronds could also help virions to penetrate the overlying glycocalyx.

Gp48-induced membrane fronds are unlikely to promote MHV-68 spread in isolation. Virion-laden vesicles appeared to travel within larger, tubulin-filled processes (Figure 5A) before fusing with the plasma membrane, thereby extending further their range of dissemination. Infected cells also frequently detached from and re-attached to dishes under time-lapse imaging (data not shown). The significance of this was unclear, but it could conceivably reflect a mechanism of spread that complements conventional virion release. A B cell-tropic pathogen such as MHV-68 can also use lymphocyte recirculation to move over large distances. And of course herpesviruses persist in mobile hosts. Thus, different mechanisms of viral spread can be brought into play depending on the scale of movement required. Gp48 functioned on a micro-scale, enhancing the spread of virions to immediately neighbouring cells. This complements larger scale spread mechanisms and so maximizes the efficiency with which MHV-68 moves within and between its hosts.

## Materials and Methods

### Plasmids

Retrovirus-expressed human CD8α [26] and ORF27 [9], and the pEGFPC2-ORF58 fusion construct [10] have been described. mRFP-ORF58 was derived from pEGFPC2-ORF58 by replacing the eGFP coding sequence with that of mRFP [27]. ORF27 (254 amino acid residues) was subcloned from pMSCV-ORF27-IRES-ZEO as a *Eco*RI/*Xho*I-restricted fragment into the *Eco*RI/*Sal*I sites of pEGFPC2. Truncation mutants encoding ORF27 residues 1-158, 1-112, 1-88, 18-88, 38-88 or 18-254 were amplified from full-length ORF27 by PCR and also cloned as *Eco*RI/*Xho*I-restricted fragments into pEGFPC2. Thus, each ORF27 derivative had eGFP attached to its cytoplasmic N-terminus. Dominant negative clones of Rac1 (Rac1N7), RhoA (RhoN19) and Cdc42 (Cdc42N17) were kindly provided by Dr. P. Digard (Division of Virology, Cambridge, U.K.). pGEX-KG-TAT-C3 was kindly provided by Dr. Michael Way (CR-UK, London, U.K.), and expressed in *E.coli* by standard methods [28]. Plasmids were transfected using lipofectamine (Invitrogen Corporation, Paisley, U.K.).

### Cell lines

BHK-21 cells, 293T cells, NIH-3T3 cells, the CD8α-transduced derivative 3T3-CD8 and the cre-transduced derivative 3T3-CRE [29] were grown in Dulbecco's modified Eagle medium (Invitrogen Corporation) supplemented with 2 mM glutamine, 100 U/ml penicillin, 100 µg/ml streptomycin and 10% fetal calf serum (PAA Laboratories, Linz, Austria).

### Viruses

MHV-68 genomic co-ordinates 78254-79298 [30] were PCR-amplified with *Age*I-restricted and *Ase*I-restricted primers and cloned into the *Age*I and *Ase*I restriction sites of pEGFP-C2-ORF58. This added a second genomic flank to the eGFP coding sequence. The flanked eGFP was then subcloned into the pST76-SR shuttle vector as a *Bam*HI/*Kpn*I fragment, using a *Bam*HI site in pEGFP-C2 and a *Kpn*I site in the *Ase*I-restricted primer. The eGFP coding sequence was then recombined into the MHV-68 BAC ORF58 locus by standard protocols [31]. An ORF27-deficient version of the eGFP-ORF58 tagged virus was made by a second round of shuttle mutagenesis, introducing stop codons into an ORF27 genomic *Sca*I site as described [9]. Each MHV-68 BAC (eGFP-ORF58, eGFP-ORF58/27^−^) was reconstituted into infectious virus by transfection into BHK-21 cells using Fugene-6 (Roche Diagnostics, Lewes, U.K.). The loxP-flanked BAC cassette was removed by passaging the virus through 3T3-CRE cells. ORF27-deficient and ORF58-deficient mutants on a wild-type MHV-68 background [9], [10] and MHV-68 with eGFP-tagged gM [19] have been described. All virus stocks were grown in BHK-21 cells. Cell debris was pelleted by low-speed centrifugation (1,000×*g*, 3 min) and discarded. Virions were then recovered from supernatants by high-speed centrifugation (38,000×*g*, 90 min), and stored at −70°C. Viruses were titered by plaque assay on BHK-21 cells [12].

### Southern blotting

DNA was extracted from virus stocks by alkaline lysis [12], digested with restriction endonucleases, electrophoresed in 0.8% agarose, and transferred to positively charged nylon membranes (Roche Diagnostics). ^32^P-dCTP-labeled probes (APBiotech, Little Chalfont, U.K.) were generated by random primer extension (Qbiogene, Bingham, U.K.). Membranes were hybridized with the probe (65°C, 18 h), washed in 30 mM sodium chloride/3 mM sodium citrate/0.1% sodium dodecyl sulfate at 65°C, and exposed to X-ray film.

### Flow cytometry

BHK-21 cells were left uninfected or infected (2 p.f.u./cell, 18 h) with eGFP-ORF58 or eGFP-ORF58/27^−^ MHV-68. The cells were then trypsinized and stained (1 h, 4°C) with mAb 6D10 (gp48), mAb 3F7 (gN) or T1A1 (gp150), plus phycoerythrin-conjugated goat anti-mouse IgG pAb (Sigma Chemical Co., Poole, U.K.). The cells were washed ×2 in PBS after each antibody incubation and analyzed on a FACSort (BD Biosciences, Oxford, U.K.).

### Electron microscopy

BHK-21 cells were infected with wild-type MHV-68 (2 p.f.u./cell, 18 h) then fixed, stained and embedded for transmission electron microscopy as described [12].

### Immunofluorescence

NIH-3T3, 3T3-CD8, BHK-21 or 293T cells on glass cover slides were infected (1 p.f.u./cell) or not with MHV-68, or transfected with plasmids. Where indicated, the cells were treated with Brefeldin A (18 µM) or Nocodazole (20 µM) for 1 h at 37°C, or with *C.difficile* Toxin B (5 ng/ml) or recombinant TAT-C3 protein (1 µg/ml) for 4 h at 37°C. All the cells were then fixed (4% paraformaldehyde, 20 min), permeabilized (0.1% Triton X-100, 15 min) and blocked (3% bovine serum albumin in PBS, 15 min). MHV-68 proteins were stained using mAbs 3F7 (gN) [21], T1A1 (gp150) [12], T8H3 (gp48), 6D10 (gp48) [10], T3B8 (gp70) [32] or MG-12B8 (ORF65 capsid component) [19], plus Alexa568-conjugated goat anti-mouse IgG pAb (Invitrogen). Actin was stained with Alexa568-conjugated phalloidin (Invitrogen). Alpha tubulin was stained with mAb, YL1/2 (Serotec, Oxford, U.K.) plus Alexa568-conjugated goat anti-rat IgG pAb (Invitrogen). CD8α was stained with mAb HIT8a (BD Biosciences) plus Alexa568-conjugated goat anti-mouse IgG pAb. EGFP-tagged and mRFP-tagged proteins were visualized directly. The cells were then mounted in ProLong Gold anti-fade reagent with DAPI (Invitrogen). For time lapse imaging, live cells were housed in a heated incubation chamber with constant CO_2_. All images were captured with a Leica SP2 confocal microscope and analysed using ImageJ.

## Supporting Information

Figure S1 MHV-68 induces ORF27-dependent membrane fronds on NMuMG epithelial cells. A. NMuMG cells were infected (1 p.f.u./cell, 16 h) with ORF27+ or ORF27- MHV-68, each with eGFP-tagged ORF58. The cells were then fixed, permeabilized and stained for the ORF65 (capsid) with mAb MG-12B8 plus Alexa568-conjugated goat anti-mouse IgG pAb. Nuclei were counter- stained with DAPI. EGFP fluorescence was visualized directly. EGFP+ membrane fronds were seen only when ORF27 was intact. B. NMuMG cells were infected with ORF27+ eGFP-ORF58-tagged MHV-68 (1 p.f.u./cell, 16 h), then fixed, permeabilized and stained for gN with mAb 3F7 plus Alexa568-conjugated goat anti-mouse IgG pAb. EGFP fluorescence was visualized directly. The punctate gN staining in more distal membrane fronds presumably corresponds to virions. C. NMuMG cells were infected as in B, then fixed, permeabilized and stained for actin with Alexa-568-conjugated phalloidin. EGFP fluorescence was visualized directly.
(5.79 MB TIF)

Figure S2 Gp150 is accessible on membrane fronds without permeabilization. NIH-3T3 cells were infected with gM-eGFP expressing MHV-68 (1 p.f.u./cell, 16 h), then either stained intact or first fixed with paraformaldehyde and permeabilized with Triton-X100. ORF65 was visualized with mAb MG-12B8 and gN with mAb 3F7. The intact cells were fixed and permeabilized after staining. EGFP-ORF58 was viewed directly and nuclei were counter-stained with DAPI. The zoomed images correspond to the boxed regions. The lack of difference between permeabilized and non-permeabilized gN staining, particularly in the punctate staining of the distal membrane fronds, argued against virions being contained within the fronds.
(7.50 MB TIF)

Movie S1 BHK-21 cells were infected with gM-eGFP-tagged MHV-68 (1 p.f.u./cell, 16 h), then imaged every 7 sec over 2.5 min. The top panel shows gM-eGFP, the bottom panel phase contrast images.
(3.14 MB MOV)

Movie S2 This is a higher magnification view of Movie S1, taken slightly later and focussing on a large cellular process and the fronds around it. The dots in the upper third of the picture probably correspond to single virions attached to distal membrane fronds. gM-eGFP tended to light up only the proximal fronds because gM is more highly expressed in virions than on the plasma membrane.
(10.58 MB AVI)

Movie S3 A single long process of an infected (gM-eGFP MHV-68, 1 p.f.u./cell, 16 h) BHK-21 cell is shown, with images captured every 10 sec over 10 min. Note the motility of the plasma membrane over this time. The fine fronds branch off from this process, as can be seen from its “hairy” phase contrast appearance. The eGFP+ dots are probably individual virions.
(6.19 MB MOV)

Movie S4 A higher magnification view of Movie S3, taken slightly later shows the mobility of the eGFP+ dots about the process to which they are attached. Both the attached vesicle in the centre of the phase contrast image and the eGFP+ dots appear to lie outside the membrane process.
(3.49 MB MOV)

Movie S5 NIH-3T3 cells were infected (0.1 p.f.u./cell, 16 h) with eGFP-ORF58-tagged MHV-68. Images were captured every 10 sec over 5 min. ORF58 is expressed more on the plasma membrane than in virions, and therefore lights up finer membrane fronds. These are seen to reach out to a neighbouring, uninfected cell. Note also the highly mobile, even pulsatile membrane of the infected cell. This was a consistent feature of MHV-68 infection.
(3.93 MB MOV)

Movie S6 NIH-3T3 cells were infected (0.3 p.f.u./cell, 16 h) with gp48-deficient eGFP-ORF58-tagged MHV-68, then imaged every 20 sec over 10 min. Note the lack of eGFP+ connections between infected and uninfected cells.
(6.23 MB MOV)

Movie S7 NIH-3T3 cells were infected (0.3 p.f.u./cell, 16 h) with gp48+ eGFP-ORF58-tagged MHV-68, then imaged every 20 sec over 10 min. Membrane fronds are seen to extend out from the central infected cell, mostly towards an uninfected cell on its lower left. These fronds grow noticeably in length over 10 min.
(5.16 MB MOV)
