# Supplementary figures and images for: A Gamma-Herpesvirus Glycoprotein Complex Manipulates Actin to Promote Viral Spread
Source: PLoS One. 2008 Mar 19;3(3):e1808. doi: 10.1371/journal.pone.0001808 (PMC2262946; doi:10.1371/journal.pone.0001808)

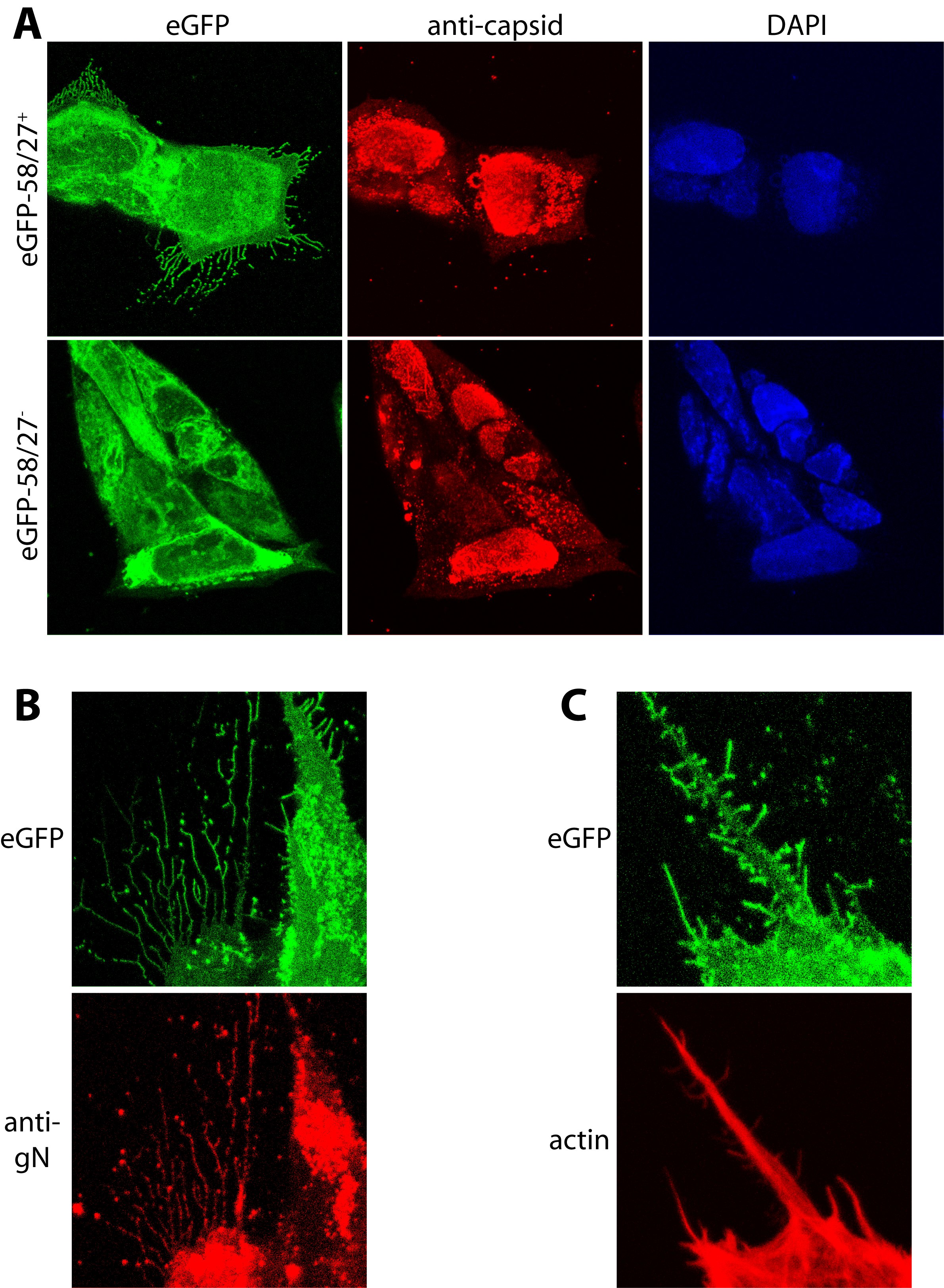

Supplement: Figure S1 — MHV-68 induces ORF27-dependent membrane fronds on NMuMG epithelial cells. A. NMuMG cells were infected (1 p.f.u./cell, 16 h) with ORF27+ or ORF27- MHV-68, each with eGFP-tagged ORF58. The cells were then fixed, permeabilized and stained for the ORF65 (capsid) with mAb MG-12B8 plus Alexa568-conjugated goat anti-mouse IgG pAb. Nuclei were counter- stained with DAPI. EGFP fluorescence was visualized directly. EGFP+ membrane fronds were seen only when ORF27 was intact. B. NMuMG cells were infected with ORF27+ eGFP-ORF58-tagged MHV-68 (1 p.f.u./cell, 16 h), then fixed, permeabilized and stained for gN with mAb 3F7 plus Alexa568-conjugated goat anti-mouse IgG pAb. EGFP fluorescence was visualized directly. The punctate gN staining in more distal membrane fronds presumably corresponds to virions. C. NMuMG cells were infected as in B, then fixed, permeabilized and stained for actin with Alexa-568-conjugated phalloidin. EGFP fluorescence was visualized directly. (5.79 MB TIF) [file pone.0001808.s001.tif]

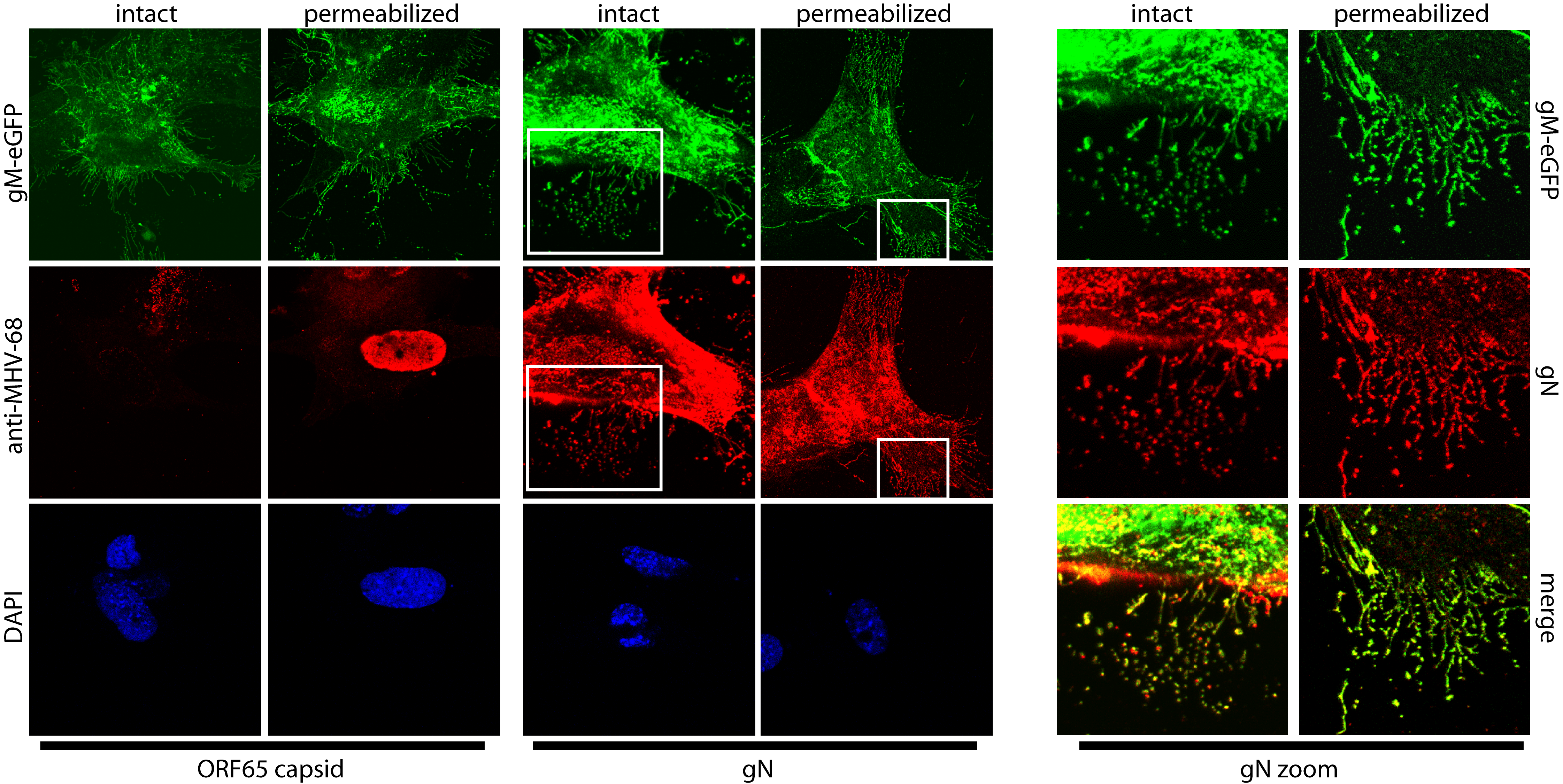

Supplement: Figure S2 — Gp150 is accessible on membrane fronds without permeabilization. NIH-3T3 cells were infected with gM-eGFP expressing MHV-68 (1 p.f.u./cell, 16 h), then either stained intact or first fixed with paraformaldehyde and permeabilized with Triton-X100. ORF65 was visualized with mAb MG-12B8 and gN with mAb 3F7. The intact cells were fixed and permeabilized after staining. EGFP-ORF58 was viewed directly and nuclei were counter-stained with DAPI. The zoomed images correspond to the boxed regions. The lack of difference between permeabilized and non-permeabilized gN staining, particularly in the punctate staining of the distal membrane fronds, argued against virions being contained within the fronds. (7.50 MB TIF) [file pone.0001808.s002.tif]
